# IL-2-based approaches to Treg enhancement

**DOI:** 10.1093/cei/uxac105

**Published:** 2022-11-18

**Authors:** Ffion Harris, Yoana Arroyo Berdugo, Timothy Tree

**Affiliations:** Department of Immunobiology, Faculty of Life Sciences and Medicine, King’s College, London, UK; Department of Immunobiology, Faculty of Life Sciences and Medicine, King’s College, London, UK; Department of Immunobiology, Faculty of Life Sciences and Medicine, King’s College, London, UK; National Institute of Health Research Biomedical Research Centre at Guy’s and St. Thomas’ National Health Service Foundation Trust, King’s College London, London, UK

**Keywords:** regulatory T cells, autoimmunity, immunotherapy, autoinflammatory disease, cytokines

## Abstract

Immune homeostasis is heavily dependent on the action of regulatory T cells (Tregs) which act to suppress the activation of many immune cell types including autoreactive conventional T cells. A body of evidence has shown that Tregs are intrinsically defective in many common autoimmune diseases, and gene polymorphisms which increase the susceptibility of autoimmune disease development have implicated the interleukin-2 (IL-2) signaling pathway as a key dysregulated mechanism. IL-2 is essential for Treg function and survival, and Tregs are highly sensitive to low levels of this cytokine in their environment. This review will revisit the rationale behind using low-dose IL-2 as a therapy to treat autoimmune diseases and evaluate the outcomes of trials to date. Furthermore, novel engineered IL-2 therapies with increased Treg specificity have shown promise in pre-clinical studies and human clinical trials for some agents have begun. Future studies will determine whether low-dose IL-2 or engineered IL-2 therapies can change the course of autoimmune and inflammatory diseases in patients.

## What are Tregs and how do they suppress?

### Tregs are vital for systemic immune regulation

The seminal paper by Sakaguchi and colleagues [1] was the first to demonstrate that CD4^+^ T-cell suspensions depleted of CD25^hi^ cells were capable of inducing multi-organ autoimmunity when transferred into athymic nude mice. These CD25^hi^ cells, later named regulatory T cells (Tregs), were identified in the human CD4^+^ T-cell compartment by several groups in 2001 [2–7]. There is now extensive evidence that Tregs play a vital role in preventing autoimmune diseases and in promoting graft tolerance in transplant patients.

Tregs constitute around 5% of CD4^+^ T cells, with the remaining 95% comprising of conventional T cells (Tconvs). Tregs can exert their suppressive function on a variety of immune cells including CD4^+^ Tconvs, CD8^+^ T cells, B cells, monocytes, dendritic cells, and natural killer cells. The mechanisms of Treg-mediated suppression have been extensively reviewed elsewhere [8, 9]. These include contact-dependent mechanisms such as expression of inhibitory immune checkpoint molecules, cytolytic molecules, secretion of suppressive cytokines such as IL-10, metabolic disruption, and sequestration of interleukin-2 (IL-2). The transcription factor Forkhead box protein P3 (FoxP3) is a master regulator of the Treg lineage [10]. The importance of FoxP3 in normal immune function is evident as the severe systemic autoimmune disease Immune Dysregulation Polyendocrinopathy Enteropathy X-linked (IPEX) syndrome is caused by mutations in the FoxP3 gene in humans [11] and a similar phenotype is observed Scurfy mice [12]. Treg survival and maintenance of their suppressive phenotype are heavily dependent on IL-2.

## IL-2 is essential for Treg survival and function

### IL-2 and its receptors

IL-2 is a 15.5kDa four-alpha-helix bundle glycoprotein comprising 133 amino acids [13] and is produced mostly by activated T cells [14] and B cells [15]. Notably, in contrast to Tconv cells which readily produce IL-2 upon T-cell receptor (TCR) engagement, Tregs cannot produce IL-2 [16].

As a member of the common gamma chain family of cytokines, IL-2 exerts its pleiotropic effects by binding its cell surface receptor complexes which are made up of 3 subunits; the IL-2 receptor α-chain (IL-2Rα; CD25), the IL-2 receptor β-chain (IL-2Rβ; CD122), and the common gamma-chain (IL-2Rγc; CD132) [17, 18]. Two IL-2 receptor complexes are capable of inducing signal transduction. Heterotrimeric association of all three subunits (IL-2Rα/β/γc) forms the high-affinity IL-2R complex (binding affinity *K*d ≈ 10 pM) which is transiently expressed on Tconv cells upon activation, but constitutively expressed by Tregs. Association of the IL-2Rβ and IL-2Rγc chains forms the intermediate affinity complex (binding affinity *K*d ≈ 1 nM) which is expressed by resting T cells and other immune cell types. The low-affinity IL-2 receptor consists of the IL-2Rα subunit alone but cannot initiate signal transduction (reviewed by [19]).

### IL-2 signaling dysfunction impairs Tregs

Experiments pre-dating the discovery of Tregs show that knocking out either the *IL2* [20], *IL2RB* [21], or *IL2RA* [22] genes in mice induces systemic inflammation and lymphoproliferation. These studies confounded previous expectations as IL-2 had historically been considered as a growth factor for T cells [23]). *IL2RA* deficiency in mice can induce Treg apoptosis leading to lethal autoimmunity, demonstrating the profound effect that defective IL-2 signaling has on Tregs [24]).

In humans, point mutations which disrupt the expression of the *IL2RA* and *IL2RB* genes can also cause autoimmune inflammatory disease [25, 26]. Tregs from CD25-deficient patients have a reduced frequency in peripheral blood and have a decreased *in vitro* suppressive capacity [27]. Moreover, Van Zeebroeck *et al.* [28] recently showed that deletion of *IL2RA* from human Tregs using CRISPR-Cas9 technology reduces their *in vitro* suppressive capacity.

IL-2 is indispensable for Treg development in the thymus and Treg survival in the periphery. *IL2RB*-knockout mice fail to produce Tregs in the thymus, however transgenic thymic expression of normal IL-2Rβ in these mice reconstitutes normal Treg development [29]). Moreover, administration of IL-2 neutralizing antibodies can deplete Tregs from the periphery in both neonatal and adult mice [30, 31]. Taken together, these studies show that functional Tregs are highly dependent on IL-2 and their dominant suppression of Tconv cells is lost upon removal of IL-2 signaling.

## The IL-2 signaling pathway

### Tregs have a specialized intracellular signaling response to IL-2

**Figure 1.**
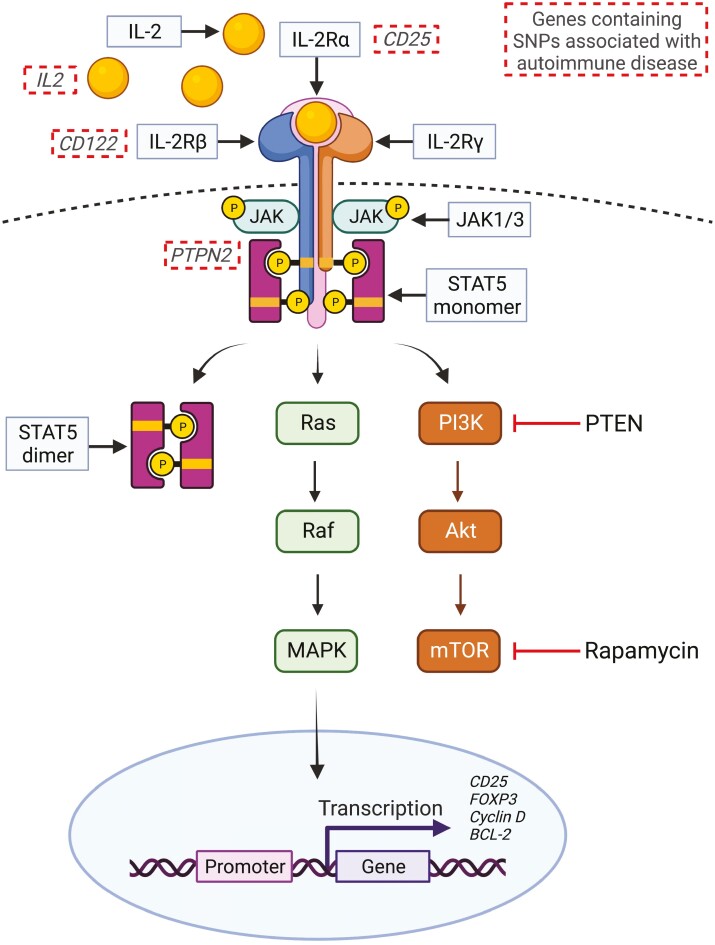
IL-2 signaling and autoimmune disease susceptibility genes. IL-2 signals through receptor complexes composed of up to 3 subunits; the IL-2 receptor-α (CD25), IL-2 receptor-β (CD122), and the IL-2 receptor-γ (CD132; common-gamma chain). In conventional T cells, engagement with the intermediate affinity receptor or the high-affinity receptor initiates signal transduction through the STAT5, Ras/Raf/MAPK, and PI3K/Akt, mTOR signaling pathways leading to expression of IL-2 response genes. Regulatory T cells express high levels of PTEN which inhibits the PI3K/Akt/mTOR pathway, leaving Tregs relatively resistant to the effects of the immunosuppressant drug Rapamycin. Genome-wide association studies have identified single nucleotide polymorphisms in several elements of the IL-2 signaling pathway which increase the risk of autoimmune disease development (dashed boxes). Figure creating using BioRender.

Signaling through the IL-2 receptor causes heterodimerization of the IL-2Rβ and γ-chain cytoplasmic domains, leading to recruitment of Janus kinase (JAK) non-receptor tyrosine kinases such as JAK1 and JAK3 (Figure 1) [32]. Importantly, the IL-2Rα chain (CD25) alone cannot initiate signal transduction. These JAK1 and JAK3 proteins in turn phosphorylate tiyrosine residues on the IL-2Rβ chain and can then propagate signal transduction through 3 pathways; the signal transducer and activator of transcription 5 (STAT5) pathway which is the dominant downstream pathway in Tregs [33], and the Ras/Raf/MAPK and the PI3K/Akt/mTOR pathways in Tconvs (reviewed in [34]). These pathways ultimately lead to expression of IL-2 target genes such as *IL2RA*, *FoxP3*, *Cyclin D2*, *Bcl-2* [35].

IL-2 signaling though the STAT5 pathway is pivotal to Treg function and its dominance in Tregs marks a major difference in IL-2 signaling between Tregs and Tconvs. Signaling through the PI3K/Akt/mTOR pathway is inhibited in Tregs through high expression of PTEN (phosphatase and tensin homolog) and this mechanism restricts Treg proliferation in response to IL-2 [36]. Inhibition of signaling through this pathway appears to be important for Treg function as activating Akt causes a loss of suppressive ability in Tregs [37]. In contrast, Tconv cells strongly proliferate in response to IL-2-mediated PI3K/Akt/mTOR signaling, and this response can be inhibited by use of the immunosuppressive drug rapamycin which targets mTOR to bring about cell cycle arrest [38, 39]. As Tregs do not utilize IL-2-mediated PI3K/Akt/mTOR signaling, these cells are resistant to rapamycin-induced hypoproliferation. Addition of rapamycin to culture media upon TCR stimulation also induces a functionally suppressive phenotype in Tconvs [40].

### IL-2 signaling in other immune cells

IL-2 receptor complexes are also expressed on other immune cells other than CD4^+^ T cells, making them responsive to IL-2. For example, IL-2 is required during initial priming of CD8^+^ T cells which are capable of expanding upon secondary antigen exposure [41]. Natural killer cells (NK) cells are known to proliferate strongly and produce cytokines in response to IL-2, including CD56^bright^ NK cells which are thought to have an immunoregulatory role [42, 43]. Additionally, IL-2 promotes activation and proliferation of type 2 innate lymphoid cells (ILC2) as these cells express high levels of CD25 [44]. In humans and mice, IL-2 administration has been shown to induce secretion of IL-5 by these cells leading to downstream eosinophilia [45].

### Tregs have a heightened sensitivity to IL-2

Tregs are highly sensitive to low levels of IL-2 in their environment, partially owing to their constitutive and high expression of the high-affinity IL-2 receptor complex (IL-2Rα/β/γc). This allows Tregs to sequester extracellular IL-2 from their environment, a process known as the IL-2 ‘sink’ suppressive mechanism, thus limiting Tconv growth while promoting a more suppressive Treg phenotype (reviewed by [46]).

However, Tregs experience IL-2-mediated STAT5-phosphorylation at around a 10-fold lower IL-2 concentration than either conventional memory T cells or T-cell blasts induced to express high levels of surface IL-2Rα, and activation of IL-2-dependent gene-responses can occur at a 100-fold lower IL-2 concentration than in Tconvs [47]. This suggests an intrinsically heightened sensitivity to IL-2 signaling which is independent of the high affinity IL-2 receptor and this may be due to IL-2 signal transduction being diverted away from the PI3K/Akt/mTOR and Ras/MAPK pathways. Together, Tregs have both a competitive advantage for IL-2 and a lower IL-2 signaling threshold. These features have formed the biological basis of low-dose IL-2 as a therapy, taking advantage of Tregs’ high responsiveness to concentrations of IL-2 which are too low to induce activation of Tconvs. Given the pivotal role that Tregs play in immune regulation, there is a strong rationale for therapeutically strengthening Tregs through IL-2 replenishment.

### Evidence of Treg dysfunction in autoimmune diseases

Thymic selection has evolved to eliminate self-reactive cells from the T cell pool. However, this an imperfect process as low frequencies of autoreactive T cells are readily detectable in healthy individuals [48]. Despite this, autoreactive T cells are maintained in a functionally suppressed state in the periphery by Tregs thus preventing the onset of autoimmune disease. Depletion of Tregs at any stage in life can lead to autoimmunity in mice [49] and evidence suggests that Treg dysfunction in humans can lead to autoimmunity.

There has been a longstanding debate in the research community as to whether Tregs from individuals with autoimmune diseases are deficient in number or function, a subject reviewed in detail elsewhere [50–52]. Conflicting studies in classical autoimmune diseases such as systemic lupus erythematosus (SLE) and rheumatoid arthritis (RA) have shown either an increase [53, 54] or a decrease [55, 56] in total Treg frequency or number when compared to disease free controls. A substantial contribution to these discrepancies is likely to be caused by the precise combination of markers used to identify Tregs in flow cytometry experiments (including CD25, CD127, FOXP3, and Helios) and the fact that these can be variably expressed by activated non-regulatory T cells, especially in the inflammatory autoimmune environment. Other factors including the tissue used for testing, different stages of disease being investigated, and selection of the comparator population are likely to further affect reproducibility.

What is perhaps more widely accepted is that Treg suppressive function is reduced compared to age matched controls in a range of different autoimmune and inflammatory diseases including SLE [57–59], RA [60, 61], multiple sclerosis [62], ankylosing spondylitis [63], systemic sclerosis [64], and type 1 diabetes (T1D) [65–68]. Studies have suggested that reduced suppression is a factor both of increased resistance to suppression by effector T cells and intrinsic changes in Tregs themselves, both of which are likely to be compounded at sites of inflammation.

Although several different mechanisms leading to Treg dysfunction have been reported, IL-2 and its signaling components are commonly associated with defective Treg function in autoimmune diseases. For example, a study from our group and collaborators demonstrated that Tregs from T1D patients can exhibit reduced responsiveness to IL-2 leading to reduced Treg frequency, loss of FoxP3 expression, and reduced suppressive function [68]. This Treg phenotype is enriched in patients harboring single-nucleotide polymorphisms (SNPs) in protein tyrosine phosphatase N2 (*PTPN2*) and similar results have been shown in healthy individuals carrying *PTPN2* SNPs [69]. *PTPN2* is one of several genes encoding proteins involved in the IL-2 signaling pathway, including *IL2* itself, which associate with T1D (Figure 1) [70, 71] RA, and Crohn’s disease [72] in genome wide association (GWAS) studies. This serves as evidence that IL-2 signaling discrepancies are causative factors in autoimmune diseases and not consequences of disease progression.

In addition to genetic factors which limit the ability of IL-2 to stabilize the Treg phenotype, environmental factors may also play a role. A state of persistent inflammation exists in most autoimmune diseases, and the inflammatory milieu driven by innate activation could reduce the functionality of Tregs. For example, several studies have demonstrated that IL-6 plays a role in inhibiting Treg function and promoting differentiation of Th17 Tconvs through altering the balance of STAT3 and STAT5 activation (reviewed by [73]). Elevated serum and tissue IL-6 have been reported in RA [74] and SLE [75]. Microbial dysbiosis is another environmental factor which is thought to influence autoimmune disease susceptibility. SLE and RA patients display characteristic patterns of altered microbiome diversity [76, 77] and probiotic treatment with protective bacterial strains such as *L. salivarius* induced anti-arthritic effects and increased the Treg:Th17 ratio in arthritis-prone mice [78].

## Low-dose IL-2 as an autoimmune disease therapy: summary of trials to date

### Low-dose IL-2 trials

Clinical trials using low doses of IL-2 aim to take advantage of Tregs’ high sensitivity to this cytokine while avoiding activation of Tconvs, thus expanding Treg numbers *in vivo* to promote immune tolerance. IL-2, also called Proleukin (brand name aldesleukin; Novartis), has been approved for use since 1992 and was first trialed at high doses as an anti-cancer drug [79]. However, a clinical benefit was observed only in unpredictable subgroups of patients and this therapy was associated with very high, sometimes lethal, toxicities. Another undesirable effect was the considerable expansion of Tregs in these cancer patients, therefore reduced doses were considered as a treatment for autoimmune and inflammatory diseases. Early studies in mouse models demonstrated a strong rationale for using low doses of IL-2 to treat T1D [80, 81].

Since the publication of the first study showing efficacy of low-dose subcutaneous IL-2 therapy in the treatment of hepatitis C-induced vasculitis [82] and graft-versus-host disease (GvHD) [83], this treatment has appeared in more than 30 different autoimmune and inflammatory disease studies and clinical trials. These include Alopecia areata, Amyotrophic lateral sclerosis (ALS), T1D, RA, ankylosing spondylitis (AS), SLE, psoriasis, Behcet’s disease, granulomatosis with polyangiitis (GPA), takayasu’s disease, Crohn’s disease (CD), ulcerative colitis (UC), autoimmune hepatitis (AIH), and sclerosing cholangitis. Brief details of treatments, biological and clinical outcomes are summarized in Table 1. These data highlight the vast range of dosing regimens that fall under the umbrella of ‘low dose IL-2’ making detailed comparison of individual studies difficult, even within a single clinical condition. In the sections that follow, we aim to draw general conclusions from these studies and highlight major areas of ongoing protocol development.

**Table 1.** Low-dose IL-2 trials. Table summarizing the results of low-dose IL-2 trials in autoimmune and inflammatory diseases to date.

Condition/publication	Clinical trial	IL-2 treatment	Biological response	Clinical measures	Clinical trial number
Alopecia areata [84]	Single-centre, uncontrolled Phase I (*n* = 5)	15 MIU/d for 5d, followed by 3 MIU/d for 5d at week 3, 6 and 9	↑ Tregs	Improved	NCT01840046
Alopecia areata [85]	Multicentre, randomized placebo-controlled	15 MIU/d for 5d, followed by 3 MIU/d for 5d at week 3, 6 and 9	↑ Total Tregs + naïve Tregs ↑ eosinophils	No changes	NCT01840046
ALS [86]	Single-centre, randomized (1:1:1) double-blind, placebo-controlled Phase IIa (*n* = 36)	3 cycles of placebo, 1 or 2 MIU/d for 5 days every 4 weeks	↑ Tregs ↑ NK cells, eosinophils, CD8^+^ T cells ↓ Monocytes	No changes	NCT02059759
Autoimmune hepatitis [87]	Pilot study (*n* = 2)	6 monthly cycles of 1 MIU/d for 5d	↑ Tregs ↓ NK cells	Improved in 1 patient (n=2)	NA
GVHD [83]	Single-centre, Phase I (*n* = 29)	03, 1 or 3 MIU/m^2^/d for 8 weeks	↑ Tregs ↑ NK cells	Improved	NCT00529035
GVHD [88]	Phase II (*n* = 16)	01-02 MIU/m^2^/d for 6-12 weeks	↑ Tregs	Improved	NCT00539695
GVHD [89]	Phase I (*n* = 21; 11 children and 10 adults)	Children 033 to 1 MIU/m^2^/d Adults 067 to 2 MIU/m^2^/d Dose escalations at weeks 2 and 4	↑ Tregs ↑ NK cells and eosinophils	Improved	NCT02318082
HCV-induced vasculitis [82]	Single-centre, uncontrolled, Phase I/II (*n* = 10)	15 MIU/d for 5 days, followed by 3 MIU/d for 5d at weeks 3, 6 and 9	↑ Tregs ↑ CD8^+^ Tregs ↑ NK cells and CD56^bright^	Improved	NCT00574652
T1DM [90]	Single-centre, uncontrolled Phase I (*n* = 9)	45 MIU/d 3×/week for 4 weeks; rapamycin 2 mg/d, followed by doses to maintain 5-10 ng/ml for 3 months	↑ Tregs ↑ NK cells and eosinophils ↓ B cells	Deteriorated	NCT00525889
T1DM [91]	Single-centre, randomized, placebo-controlled, double-blind Phase I/II (*n* = 24)	033, 1 or 3 MIU/d for 5 days	↑ Tregs ↑ eosinophils ↓ B cells	No evaluated	NCT01353833
T1DM [92]	Single-centre, randomized, placebo-controlled, double-blind, Phase I/II (*n* = 24 same patients [91])	033, 1 or 3 MIU/d for 5 days	↑ Tregs + memory Tregs ↑ CD8^+^ Tregs ↑ NK cells and CD56^bright^ ↓ B cells	Not evaluated	NCT01353833
T1DM [93]	Single-centre, uncontrolled, adaptative dose-finding Phase I/II (learning phase n=10, adaptative phase *n* = 30)	Learning phase: single dose either 0004, 016, 060, 1 or 15 MIU/m^2^; adaptative phase: single dose to achieve Treg increases of 10%–20% from baseline	↑ Tregs ↑ NK cells CD56^bright^ ↓ CD8^+^ T cells, NK and B cells	Not evaluated	NCT01827735
T1DM [94]	Multicentre, randomized, double-blind, placebo-controlled, dose-finding Phase I/II (*n* = 24 children)	Placebo, 0125, 025 or 05 MIU/m^2^/d for 5 days followed by fortnightly for 1 year	↑ Tregs ↑ Eosinophils	No changes	NCT01862120
Polymyositis/Dermatomyositis [95]	Single-centre, controlled Phase I/II (*n* = 147; 116 ST, 31 ST + IL-2)	05 MIU/d for 5 days	↑ Tregs ↑ CD8^+^ T, Th1, Th2, Th17 Tconvs and B cells	Improved	
Primary Sjögren’s syndrome [96]	Single-centre, controlled Phase I/II (*n* = 190; 91 ST, 99 ST + IL-2)	1 MIU/d for 5 days	↑ Tregs ↑ Th17 Tconvs cells	No changes	
Psoriatic arthritis [97]	Single-centre, uncontrolled Phase I/II (*n* = 201; 106 HC, 73 ST, 22 ST + IL-2)	05 MIU/d for 5 days	↑ Tregs ↑ Th1, Th2, Th17 Tconvs	Improved	
Rheumatoid arthritis [98]	Single-centre, Phase I/II (*n* = 988; 100 HC, 655 ST, 233 ST + IL-2)	1 MIU/d for 5 days	↑ Tregs ↑ Th1, Th2 and Th17 Tconvs	Improved	
SLE [99]	Pilot study (*n* = 1 ST + IL-2)	4 cycles between 15 and 3 MIU/d for 5 days; separated by 9-16 days	↑ Tregs	Improved	NA
SLE [100]	Single-centre, uncontrolled Phase I/IIa (*n* = 38)	3 cycles of 1 MIU every other day for 2 weeks followed by a 2-week break	↑ Tregs ↓ Tfh and Th17 Tconvs	Improved	NCT02084238
SLE [101]	Single-centre, uncontrolled Phase I/II (*n* = 5)	15 MIU/d for 5 days	↑ Tregs ↑ CD56^bright^ NK cells	Not evaluated	
SLE [102]	Single-centre, uncontrolled, dose-adaptation Phase I/IIa (*n* = 12 ST+ IL-2)	4 cycles between 075 and 3 MIU/d for 5 days separated by 9-16 days	↑ Tregs ↑ eosinophils ↓ Tfh cells and B cells	Improved	DRKS00004858
SLE [103]	Single-centre, controlled Phase I/II (*n* = 30; 12 ST and 18 ST + IL-2)	3 cycles of 1 MIU every other day for 2 weeks followed by a 2-week break	↑ Tregs	Improved	
SLE [104]	Single-centre, uncontrolled Phase I/II (*n* = 120; 70 HC, 50 rapamycin + IL-2)	100 WIU 3-5 days/month combined with rapamycin 05 mg every other day for 24 weeks	↑ Tregs		
SLE [105]	Single-centre, randomized, doble-blind, placebo-controlled Phase II (*n* = 60)	3 cycles of 1 MIU every other day for 2 weeks followed by a 2-week break	↑ Tregs ↑ NK cells and CD56^bright^	Improved	NCT02465580
11 autoimmune diseases: RA, AS, SLE, psoriasis, Behcet’s disease, GPA, takayasu’s disease, CD, UC, AIH and sclerosing cholangitis [106]	Multicentre, uncontrolled Phase I/IIa (*n* = 46; RA = 4, AS = 10, SLE = 6, psoriasis = 5, Behçet’s disease = 2, GPA = 1, Takayasu’s disease = 1, CD = 7, UC = 4, AIH = 2, sclerosing cholangitis = 4	1 MIU/d for 5 days, followed by fortnightly injections for 6 months	↑ Tregs ↑ CD56^bright^ NK cells and eosinophils	Improved in AS, UC, SLE and psoriasis.	NCT01988506
IL-2 combination therapies					
T1D	Single-centre, randomized, Phase II (*n* = 24) Not yet recruiting	Cyclosporin orally 35 mg/kg twice a day for 2 months following by IL-2 1MIU/d for 5 days and then every week until 1 year			NCT05153070
T1D	Single-centre, randomized, Phase I/II (*n* = 45) Not yet recruiting	Anti-thymocyte globulin 25 mg/kg in 2 infusions, 05 and 2 mg/kg at days 1 and 2 Adalimumab 50 mg/ month, for 1 year IL-2 1 MIU/d for 5 days (days 10-14) and then every 2 weeks, for 52 weeks Exenatide 2 mg SC weekly up to 52 weeks			NCT02586831
RA [107		50 WIU IL-2 per day for a 5-day course with or without 160 mg Tocilizumab at the dosage of 160 mg at days 1-3	↑ Treg:Th17 ratio	Improved	
T1D [90]	Open-label Phase I	2-4 mg/day of oral rapamycin orally for 3 months in combination with 45 MIU of IL-2 three times per week for 4 weeks	↑ Tregs ↑ NK cells and eosinophils	Deterioration of β-cell function	NCT00525889
SLE [104]		100 WIU of IL-2 for 3 to 5 days/month in combination with 05 mg of oral rapamycin every other day	↑ Tregs, ↑ Treg:Th17 ratio	Improved	
T1D [108]	Open-label, dose-escalating, Phase I	One infusion of autologous polyclonal Tregs in combination with 5-day courses of either 033 or 1 MIU of IL-2	↑ Tregs, ↑ NK cells, ↑ MAIT cells, ↑ MAIT cells, CD8^+^ T cells	Deterioration of β-cell function	NCT02772679

### Low-dose IL-2 expands Tregs

The simplest and most uniform immunological outcome in all trials is an increase in the frequency and/or absolute number of Tregs in peripheral blood which was observed in almost all studies of low-dose subcutaneous IL-2 therapy, with the unique exception of a study in alopecia areata patients [84] where Tregs were recruited to and significantly increased within scalp skin lesions but not in the blood. Furthermore, most trials to date have measured expansion of Tregs only in peripheral blood without considering homing of Tregs to affected tissues. Access to tissue from the site of inflammation is major limitation in diseases such as T1D in which serious complication have been reported following biopsy of the pancreas (reviewed by [109]). However, site-specific Treg expansion should be considered as an informative outcome measure for low-dose IL-2 trials in diseases where accessing affected tissue carries low risk. Indeed, a study by [110] showed a significant reduction in Tregs (% of CD4^+^ T cells) infiltrating cutaneous lupus erythematosus skin lesions. As many low-dose IL-2 trials in SLE patients have reported improvement in skin rashes and alopecia [100, 102], it would be highly informative for future studies to consider measuring tissue-specific Treg expansion where possible.

### Treg dose selection, response kinetics, and expanded Treg phenotype

IL-2 is known to have a very short half-life in human blood and so frequent dosing regimens are necessary [111]. Pre-clinical animal models are of little use in determining appropriate IL-2 doses for human trials as murine Tregs require much higher doses to stimulate *in vivo* expansion (as reviewed by [112]). This has resulted in a huge variation in individual and cumulative IL-2 doses, dosing frequency, and duration of IL-2 treatment between different studies making direct comparisons challenging. Overall, peripheral Tregs have been shown to increase in a dose and time-dependent manner and successive treatment cycles may have residual, cumulative effects [92]. In early studies, relatively high doses (1–3 MIU/m^2^) were used daily for prolonged periods of time resulting in a large increase in Treg frequency and number [82] but were associated with increases in other cell populations including effector CD4 T cells and NK cells. Subsequent studies suggested less frequent treatment may be optimal for maintaining selective Treg expansion resulting in protocols of daily cycles on 3–5 consecutive days followed by a rest period before repeating cycles [84, 86] or protocols based on an induction period of more intensive treatment followed by a maintenance phase [106]. Many of these protocols result in expansion of the number and frequency of Treg to a level that far exceeds normal ranges seen in disease free individuals raising concerns of generalized immunosuppression. An alternative approach to optimize expansion of Tregs within physiological levels, whilst limiting effects on other immune cells has been taken by researchers in T1D who have conducted a series of experimental medicine studies aimed at optimizing first the dose and then the frequency of administration using a response-adaptive trial design [93]. These studies identified a relatively low dose of 0.26 × 10^6^ IU/m^2^ every 3 days was sufficient to maintain a 30% increase in Tregs with minimal effects on effector T cells [113] and is currently being tested in a Phase 2 clinical trial. In summary, the optimal regimen remains unclear and is likely to vary between conditions.

Many studies have performed detailed analysis of the phenotype and function of Tregs expanded by therapy. Most studies reported that *in vivo* expanded Tregs were skewed towards a memory phenotype displaying features characteristic of enhanced activation (higher expression of CD25, GITR, CTLA-4, Ki67, Helios, CD39, CD45R0, and lower CCR7) and have a higher suppressive capacity after the treatment [86, 92, 93, 101] Contrastingly, studies in GvHD showed a delayed proliferative response of naïve CD45RA^+^Ki67^+^ Tregs in response to treatment [114] and this was accompanied by an increase in recent thymic emigrant (RTE) Tregs (CD45RA^+^CD31^+^) after around 16 weeks of treatment which was especially prominent in pediatric patients [89]. This suggests that low-dose IL-2 may have the tissue-specific effect of increasing Treg thymic output and this may be of benefit as RTE Tregs were significantly lower at baseline in the GvHD patient cohort. Moreover, a recent study in alopecia areata patients noticed an increase in frequency and numbers only in the naïve (CD45RA^+^) Treg subset; however, no clinical benefit was derived from this therapy [85]. A trial comparing the effect of low-dose IL-2 across 11 autoimmune diseases found an increase in both memory and naïve Treg subsets [106] It remains to be seen whether dosing and treatment regimen can influence the phenotype of expanded Tregs *in vivo.*

### Effect of low-dose IL-2 on other immune cells

As discussed above, many immune cell types, and some epithelial cells, express the IL-2 receptors. Although efforts have been made through dose selection to specifically target Tregs, and minimize activation of Tconvs, many other immune cell types have been shown to expand in response to low-dose IL-2 therapy. These most commonly include NK cells, of which the CD56^bright^ subset appears to be particularly sensitive to treatment regardless of disease background due to their expression of CD25 [82, 92, 93, 101, 105] and these cells are thought to have immune regulatory properties. An increase in eosinophils is frequently observed which is likely a consequence of the ILC2-IL-5-eosinophil axis [45]. Both Todd *et al.* [93] and Rosenzwajg *et al.* [106] reported increased eosinophilia in patients with high eosinophil counts at baseline, meaning future patients could be stratified for risk of eosinophilia. Other common changes in cell frequencies include changes in subsets of Tconv helper subset frequencies [95–97], and reduced T follicular helper (Tfh) cell counts [100, 102] which could be linked to reduced CD19^+^ B cell counts (Table 1). Furthermore, a recent study in SLE reported a reduction in circulating anti-double-stranded DNA antibodies [105] in treated patients suggesting autoreactive B cells may also be inhibited by this therapy. Finally, a marked increase in regulatory CD8^+^CD25^+^ T cells has been recorded by some groups [82, 92], supporting the notion that this therapy skews towards a regulatory milieu which may induce suppressive properties in many immune cell types.

### Therapeutic benefit

Many studies to date have shown an improvement in disease biomarkers during the course of low-dose IL-2 therapy, as measured by various scoring systems such as SLE responder index [100], disease activity score in psoriatic arthritis [97] and severity of alopecia tool [84], or metabolic parameters such as c-peptide decline in T1D [94] and liver enzyme and serum IgG levels in autoimmune hepatitis [87]. Despite this, very few trials to date have been designed to test the clinical efficacy of this therapy by the gold standard method of double blind, placebo-controlled trials with a pre-specified clinical efficacy outcome and no phase III trials have taken place to date.

Of the double blind, placebo-controlled trials conducted to date which have assessed clinical efficacy [85], and [94] did not see significant improvements in treated vs placebo groups in alopecia areata and T1D patients, respectively. However, these studies were small and therefore not powered to assess efficacy in large numbers of patients. A range of Phase II studies which aim to assess clinical efficacy are underway [105]. reported no significant improvement in SLE-4 score in treated vs placebo at 12 weeks (primary endpoint), but this did reach significance by week 24. Therefore, despite the robust Treg biological outcomes in every trial, clinical efficacy is yet to be proven however low-dose IL-2 continues to show promise as a future therapy.

### Heterogeneous responsiveness and clinical response predictors

There is a noteworthy, heterogeneous response of Tregs to IL-2 between individuals, highlighting the importance of monitoring dose-response rates during treatment [91] and suggesting dose personalization may be important to optimize therapy. The clinical and biological response to IL-2 is likely to be complicated by disease duration and staging. In individuals with active and ongoing inflammation, response to therapy may be expected to be different to at risk individuals or patients in quiescent stages of disease. Similarly, the age of the recipient and state of thymic Treg output and differentiation status in the periphery is likely to affect responsiveness. Indeed, in clinical trials of low dose IL-2 in cGVHD, children achieved a higher Treg: Tconv ratio and better clinical responses compared to adults, suggesting that age-dependent intrinsic differences play an important role [89]. The cytokines soluble-IL-2Rα and VEGFR2 have been identified as clinical response predictors, distinguishing responders from non-responders to the therapy [94].

### Safety and adverse events

Local reactions at injection sites and flu-like symptoms are the most commonly reported adverse events with no serious adverse events. However, IL-2 dose escalation above 1 MIU/day is not well tolerated in some patients and is associated with increased NK cell expansion and increased side-effect frequency [83, 91]. In addition to the promising safety and efficacy results to date, further trials are needed to validate long-term positive outcomes for autoimmune and inflammatory disease patients.

### Combination therapies

Due to its positive impact on Tregs, low-dose IL-2 therapy has been trialed in combination with other immune-modulating or cellular therapies with an aim to restore immune regulation. A small study tested the efficacy of low-dose IL-2 therapy in combination with the anti-IL-6 receptor antibody tocilizumab in RA patients [107] as IL-6 is known to increase the Th17:Treg ratio and is a driver of this disease (Reviewed by [115]). This combination resulted in expansion of Tregs but not Th17 Tconvs, and reduced pain and swelling symptoms.

Combining low-dose IL-2 with other therapies which aim to “de-bulk” the Tconv pool by either removing or preventing the expansion of these cells is another strategy being considered. For example, a trial administering a short treatment of cyclosporin followed by low-dose IL-2 is currently recruiting newly diagnosed T1D patients (NCT05153070). Rapamycin (sirolimus) can limit Tconv proliferation in response to IL-2 through inhibiting mTOR (as described above) and also induce a suppressive phenotype in conventional CD4 T cells. A combination of IL-2 and rapamycin treatment has previously been shown to act synergistically in preventing the onset of β-cell destruction and dysglycaemia in non-obese diabetic (NOD) mice [116]). However in T1D patients, this combination therapy expanded Tregs but also caused a significant decline in β-cell function [90]. This was thought to be caused by either the rapamycin alone, or the treatment regimen in which low-dose IL-2 was given <2 weeks before the rapamycin. In contrast, this combination therapy showed efficacy in restoring Th17:Treg balance and reducing clinical disease scores in SLE patients [104]. Finally, a recent study by [108] was the first to trial low-dose IL-2 in combination with adoptive Treg therapy to treat T1D, however, this too failed to reach its secondary outcome measure of a reduction in c-peptide decline. Therefore, the clinical outcome of combination therapies are more variable depending on the disease background of treated patients.

## Beyond wild-type IL-2

### Overcoming the challenges of low-dose IL-2 therapy

**Figure 2.**
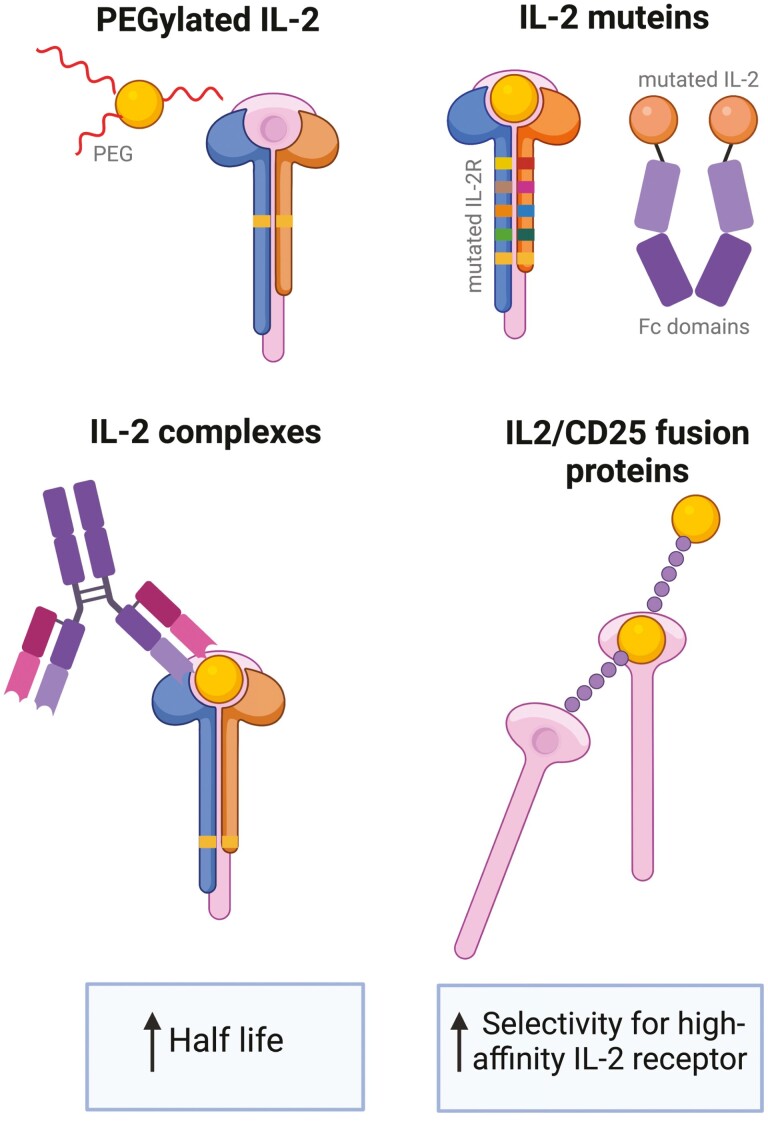
IL-2 engineering approaches. Several IL-2 technologies have been developed which act to increase the affinity of IL-2 to the high-affinity receptor complex, thus selectively expanding Tregs, and to increase its serum half-life in comparison to wild-type IL-2. Firstly, IL-2 can undergo PEGylation by attaching polyethyleneglycol chains to its surface. IL-2 muteins are generated by introducing point mutations into either the IL-2 protein or its receptor. IL-2 complexes are composed of IL-2 bound to either human or murine antibodies which act to block or conformationally alter binding sites for the IL-2 receptor-β (CD122). Finally, fusion proteins of IL-2 and the IL-2 receptor-α can also increase Treg selectivity and half-life. Figure created using BioRender.

Despite the early success of low-dose IL-2 trials in autoimmune diseases to date, there are two main limitations of this therapy as highlighted above: the short half-life of IL-2 requires frequent dosing that is low enough in individual and cumulative dosage to avoid Tconv activation, and off-target proliferative effects on other immune cells expressing IL-2 receptors. These drawbacks have inspired the development of new IL-2-engineering approaches which aim to increase the half-life of IL-2 and its specificity towards the high-affinity IL-2 receptor (Figure 2).

### IL-2 muteins

This novel technology works by introducing mutations into the IL-2 and/or IL-2R proteins to alter their affinity towards certain IL-2 receptor complexes (Figure 1). This technique was originally used to develop an IL-2 ‘superkine’ with an elevated binding affinity for IL-2Rβ (CD122) which improved *in vivo* anticancer responses [117]. Conversely, a study by Peterson *et al.* [118] showed that a novel IL-2 mutein/fusion protein IgG-(IL-2N88D)_2_ preferentially expanded Tregs *in vivo* in cynomolgus monkeys and humanised mice. Coupling of the IL-2N88D mutein to human IgG increased its *in vivo* serum half-life. A Phase I clinical trial of this drug (RO7049665) was completed in 2019; however, Phase II trials testing its clinical benefit in ulcerative colitis and autoimmune hepatitis have since been terminated early due to lack of efficacy (Table 2).

**Table 2:** Engineered IL-2 trials

IL-2 engineering	Agent	Study phase	Disease background	Trial number	Progress
Mutein	Efavaleukin alfa	Phase I/II	GvHD	NCT03422627	Ongoing
Mutein	Efavaleukin alfa	Phase I	SLE	NCT03451422	Ongoing
Mutein	Efavaleukin alfa	Phase II	SLE	NCT04680637	Ongoing
Mutein	Efavaleukin alfa	Phase II	UC	NCT04987307	Ongoing
Mutein	Efavaleukin alfa	Phase II	RA	NCT03410056	Terminated early
Mutein	Efavaleukin alfa	Phase I	Healthy	NCT04987333	Ongoing
Mutein	RG-7835	Phase II	UC	NCT03943550	Terminated early
Mutein	RG-7835	Phase I	Healthy	NCT03221179	Completed—no results posted
Mutein	RG-7835	Phase II	AIH	NCT04790916	Terminated early as no efficacy shown in RA trial
Mutein	CC-92252	Phase I	Healthy/psoriasis	NCT03971825	Terminated—did not meet progression criteria
PEGylated IL-2	NKTR-358	Phase I	Healthy	NCT04380324	Completed—no results posted
PEGylated IL-2	NKTR-358	Phase I	SLE	NCT03556007	Completed—no results posted
PEGylated IL-2	NKTR-358	Phase II	SLE	NCT04433585	Ongoing—recruiting
PEGylated IL-2	NKTR-358	Phase I	Psoriasis	NCT04119557	Completed—no results posted
PEGylated IL-2	NKTR-358	Phase I	Eczema	NCT04081350	Active—not recruiting
PEGylated IL-2	NKTR-358	Phase I	Healthy	NCT04133116	Completed—no results posted
PEGylated IL-2	NKTR-358	Phase I	UC	NCT04677179	Ongoing—recruiting

Table summarizing the completed and ongoing clinical trials using engineered IL-2 agents for the treatment of autoimmune diseases.

Recent studies using murine muteins in mouse model systems have showcased the wide therapeutic scope and selectivity of mutein therapies which could potentially be translated to humans. A study by Khoryati *et al.* [119] showed that Fc.Mut24, an IL-2 mutein fused to an IgG2a Fc domain, induced potent and specific Treg expansion, an enrichment of Tregs in the pancreas, and resolution of diabetes in NOD mice. Additionally, FC.Mut24 has shown benefit in mouse models of haemophilia (HaemA mice) [120] which are prone to formation of neutralising antibodies towards topical Factor VIII therapy. FC.Mut24 and subsequent Factor VIII gene therapy preferentially expanded Tregs and prevented anti-Factor VIII inhibitor formation in these mice [121]. have also demonstrated that antigen specific Tregs can be expanded *in vivo* using muteins such as these.

IL-2 mutein technology has been further developed through orthogonal IL-2/IL-2R pairs which cannot bind their native wild-type counterparts, giving a highly selective Treg activation system. In a murine mixed haematopoietic chimerism model, heart allograft acceptance and Treg expansion was significantly improved following adoptive transfer of orthogonal-CD122 transduced Tregs and administration of its paired orthogonal IL-2 [122].

To date, no IL-2 mutein therapies have been approved for use in humans but many clinical trials are underway testing their efficacy in classic autoimmune diseases. Efavaleukin alfa (Amgen) is currently in Phase I and Phase II trials (Table 2) testing this agent in healthy individuals, SLE, GvHD, ulcerative colitis, and rheumatoid arthritis, the latter of which has been terminated early.

### PEGylation and IL-2 complexes

PEGylation increases the half-life of IL-2 and its selectivity for intermediate or high affinity complexes through attaching polyethylene glycol (PEG) chains to lysine residues on its surface. Dixit *et al.* [123] demonstrated that the PEGylated IL-2 molecule NKTR-358 has an increased selectivity for the high-affinity IL-2 receptor complex. When administered to cynomolgus monkeys, an increase in Treg expansion and *in vitro* suppressive capacity was observed. Furthermore, this treatment halted disease progression in a murine model of SLE.

Multiple clinical trials are underway using NKTR-358 (LY3471851; Nektar therapeutics; Eli Lilly) to treat human autoimmune and inflammatory diseases such as SLE, Psoriasis, Eczema, and UC (Table 2). Preliminary results from the SLE study (NCT03556007) have shown selective expansion of Tregs and a preliminary reduction in SLE clinical scores in some patients [124].

Forming complexes between IL-2 and anti-IL-2 antibodies serve to increase its half-life and can also block CD25 or CD122 binding sites on the IL-2 protein. When bound to IL-2, the murine mAb JES6-1A12 causes a conformational change which disrupts the interaction between wild-type IL-2 and CD122/CD132. IL-2-JES6-1A12 selectively expanded Tregs and improved symptoms in mouse models of asthma [125], experimental autoimmune encephalomyelitis (EAE [126];), transplantation [127], myasthenia gravis [128], and SLE [129]. IL-2-5344 has also shown efficacy in suppressing experimental food allergy in mice [130], and IL-2-5111.2 favored the expansion of Tregs in humanized mice and halted progression of T1D, EAE, and GvHD [131]. Furthermore, the human IL-2 complex IL-2-UFKA-20 preferentially expands Tregs in non-human primates promising good translatability for this complex [132].

### IL-2 fusion proteins

Malek and colleagues were the first to develop an IL-2-CD25 fusion protein with selectivity for the high affinity IL-2 receptor complex and an increased half-life [133]. At lower doses than WT IL-2, this fusion protein has been shown to decrease the occurrence of diabetes in NOD mice [134] and decreased symptom severity in SLE mouse models [135]. A recent study has also showed that this fusion protein can induce a heightened Treg proliferative response compared to recombinant IL-2 [136].

In summary, IL-2 engineering approaches have shown promise in overcoming some of the challenges arisen from low-dose IL-2 therapies. However, further human trials are needed to assess the impact of drawbacks associated with these novel therapies. These include the need for higher much higher doses of agents which reduce signaling through the IL-2Rβ (CD122), potential immunogenicity of muteins, the hypothesized potential for non-covalently linked complexes to release free IL-2 *in vivo* (e.g. PEGylated IL-2), and off target effects on other cells which express the high-affinity IL-2 receptor.

## Conclusion

To conclude, there is a strong rationale for targeting Tregs using low doses of IL-2 to treat autoimmune diseases given the evidence from GWAS studies, mechanistic *in vitro* Treg studies, and experiments in animal models. Clinical trials to date have proven that low doses of IL-2 are safe and universally expand Tregs across a range of autoimmune diseases. Therefore, it is clear that this drug is efficient at targeting Tregs; however, its specificity for Tregs is not optimal. Despite this, there are suggestions from some trials that other cell targets may contribute to efficacy such as Tfh cells, auto-antibody producing B cells, CD56^Bright^ NK cells, and the thymus. Future clinical trials designed to evaluate clinical efficacy will shed light on whether this therapy can induce remission or halt the progression of autoimmune diseases such as SLE, GvHD, and T1D. These should also evaluate key unanswered questions such as whether expanded Tregs infiltrate disease-relevant tissues, how efficacy is affected by time-since disease onset, and would this be a financially viable treatment option for all autoimmune disease patients? Finally, IL-2 engineering approaches have successfully overcome some of the limitations of wild-type IL-2 such as increasing the half-life and specificity of IL-2 towards the high-affinity IL-2 receptor complex. Several clinical trials are underway to determine whether these drugs present an improved therapeutic avenue for autoimmune diseases than wild type IL-2 to restore immune regulation.

## Data Availability

Not applicable.

## Abbreviations

AIH: autoimmune hepatitis
ALS: amyotrophic lateral sclerosis
AS: ankylosing spondylitis
CD: Crohn’s disease
EAE: experimental autoimmune encephalomyelitis
FoxP3: Forkhead box protein P3
GPA: granulomatosis with polyangiitis
GvHD: graft versus host disease
GWAS: genome wise association
IL-2: interleukin 2
IL-2Rα: IL-2 receptor α-chain
IL-2Rγc: IL-2 receptor gamma-chain
IL-2Rβ: the IL-2 receptor β-chain
ILC2: type 2 innate lymphoid cells
IPEX: immune dysregulation polyendocrinopathy enteropathy X-linked syndrome
JAK: Janus kinase
NK cell: natural killer cell
NOD: non-obese diabetic
PEG: polyethylene glycol
PTEN: phosphatase and tensin homolog
PTPN2: protein phosphatase N2
RA: rheumatoid arthritis
RTE: recent thymic emigrant
SLE: systemic lupus erythematosus
SNP: single-nucleotide polymorphism
STAT5: signal transducer and activator of transcription 5
T1D: type 1 diabetes
Tconvs: conventional T cells
TCR: T cell receptor
Tfh: T follicular helper
Tregs: regulatory T cells
UB: ulcerative colitis
